# Supplementary material for: Development of a circHIPK3-based ceRNA network and identification of mRNA signature in breast cancer patients harboring BRCA mutation
Source: PeerJ. 2023 Jul 5;11:e15572. doi: 10.7717/peerj.15572 (PMC10329424; doi:10.7717/peerj.15572)
Supplement: Supplemental Information 1 [file peerj-11-15572-s001.pdf]

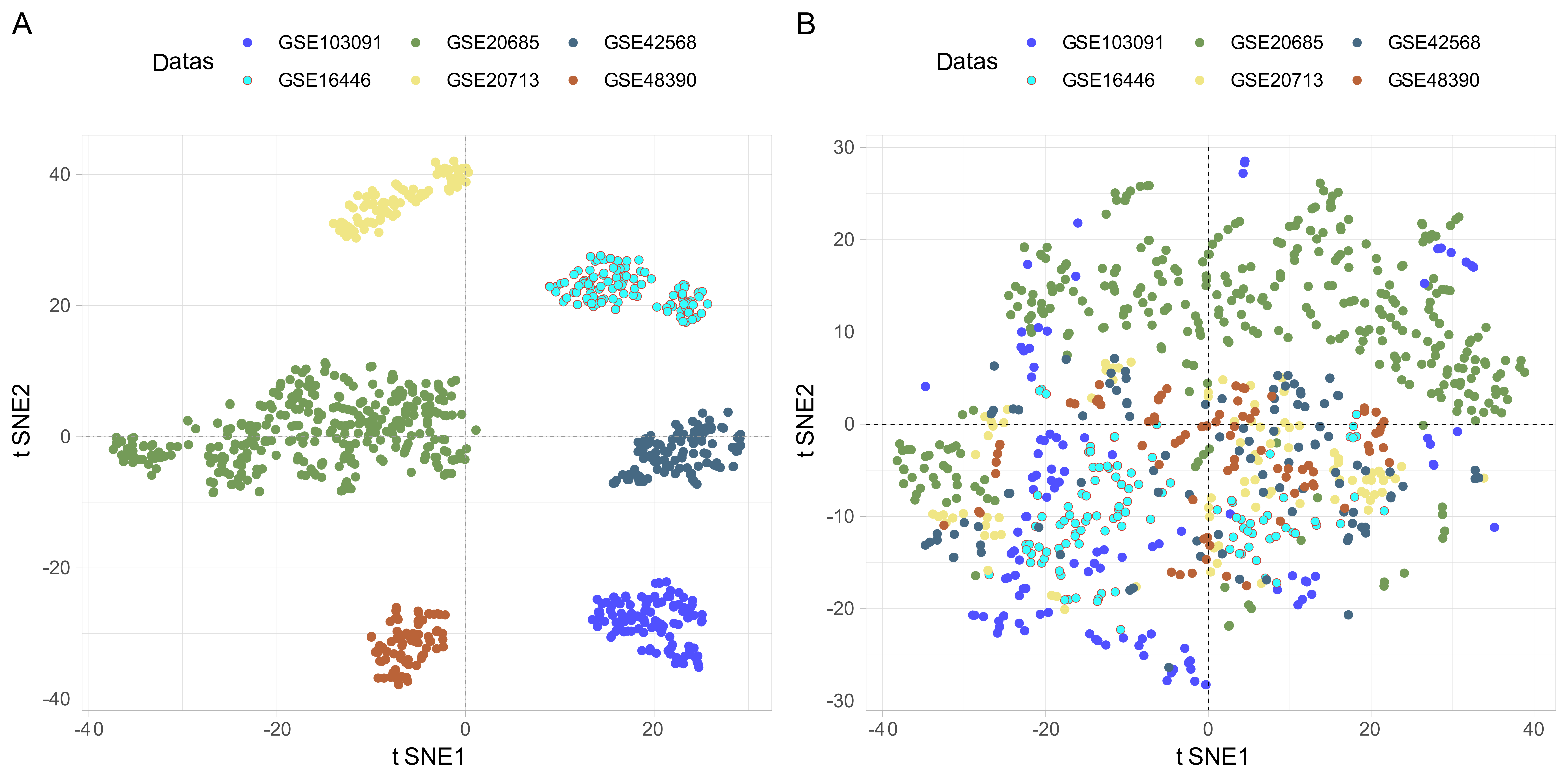
A
Datas
GSE103091
GSE20685
GSE42568
GSE16446
GSE20713
GSE48390
40
20
0
-20
-40
t SNE2
-40
-20
0
20
t SNE1
B
Datas
GSE103091
GSE20685
GSE42568
GSE16446
GSE20713
GSE48390
30
20
10
0
-10
-20
-30
t SNE2
-40
-20
0
20
40
t SNE1
