# Supplementary figures and images for: Development of a circHIPK3-based ceRNA network and identification of mRNA signature in breast cancer patients harboring BRCA mutation
Source: PeerJ. 2023 Jul 5;11:e15572. doi: 10.7717/peerj.15572 (PMC10329424; doi:10.7717/peerj.15572)

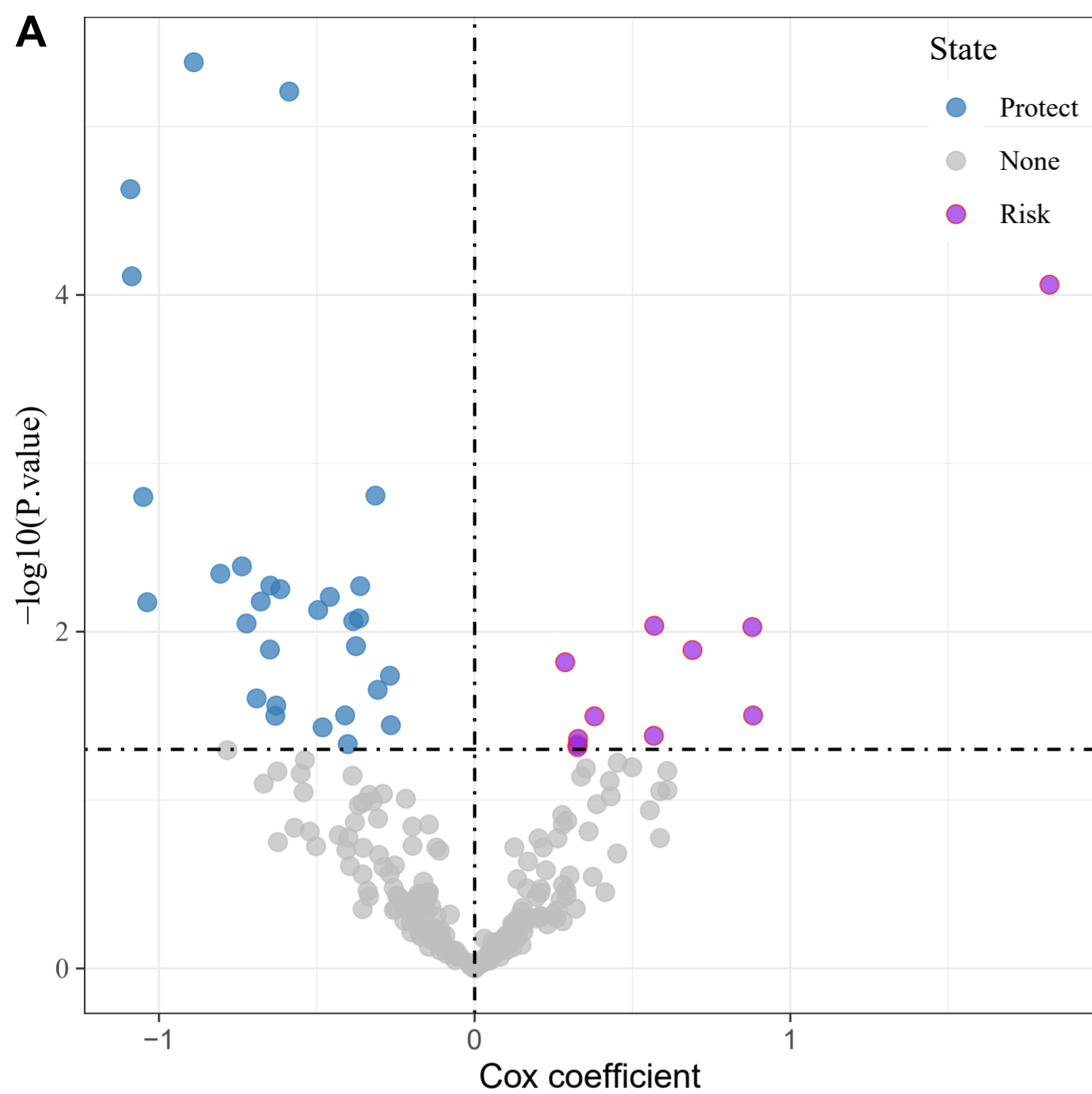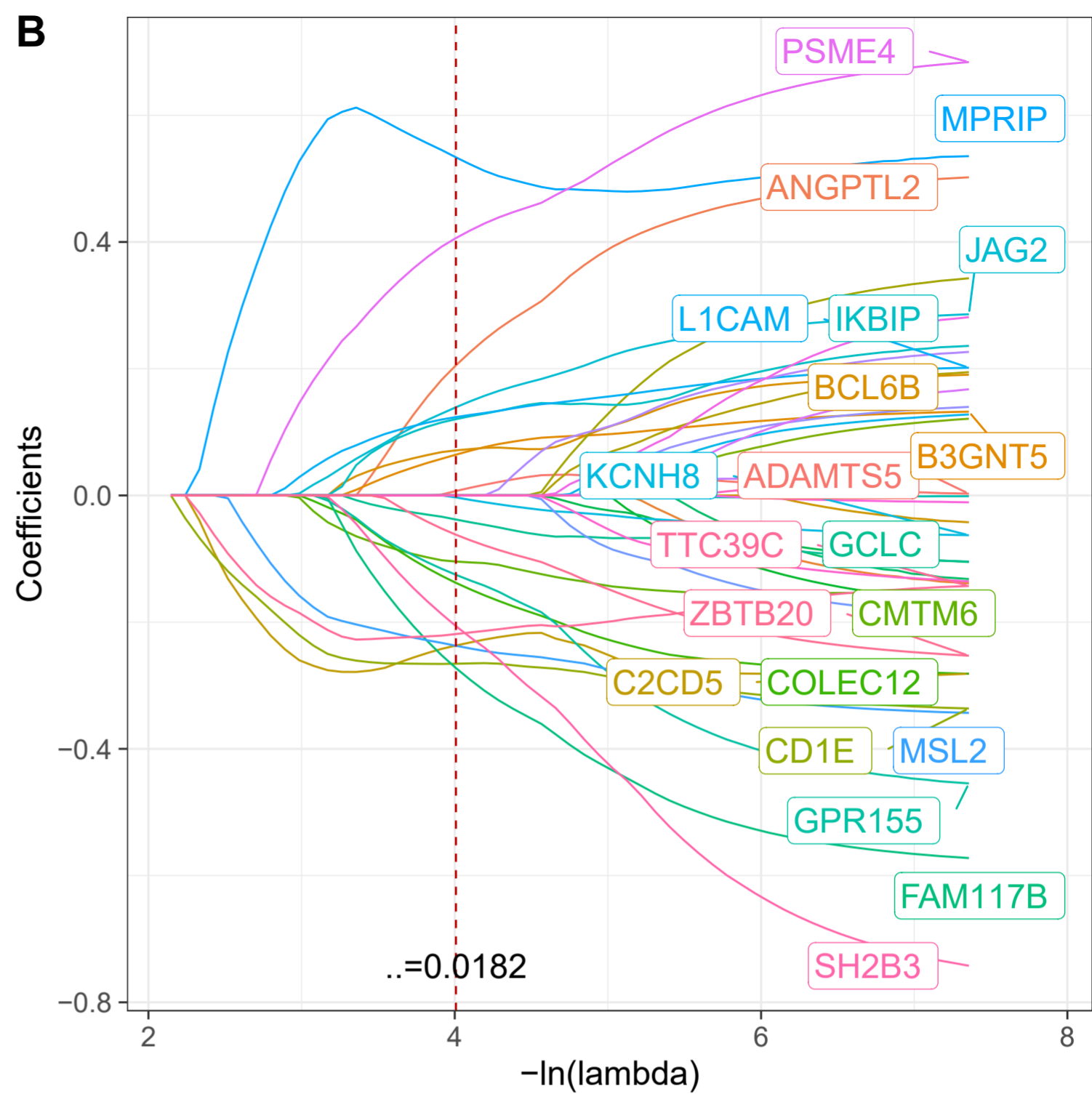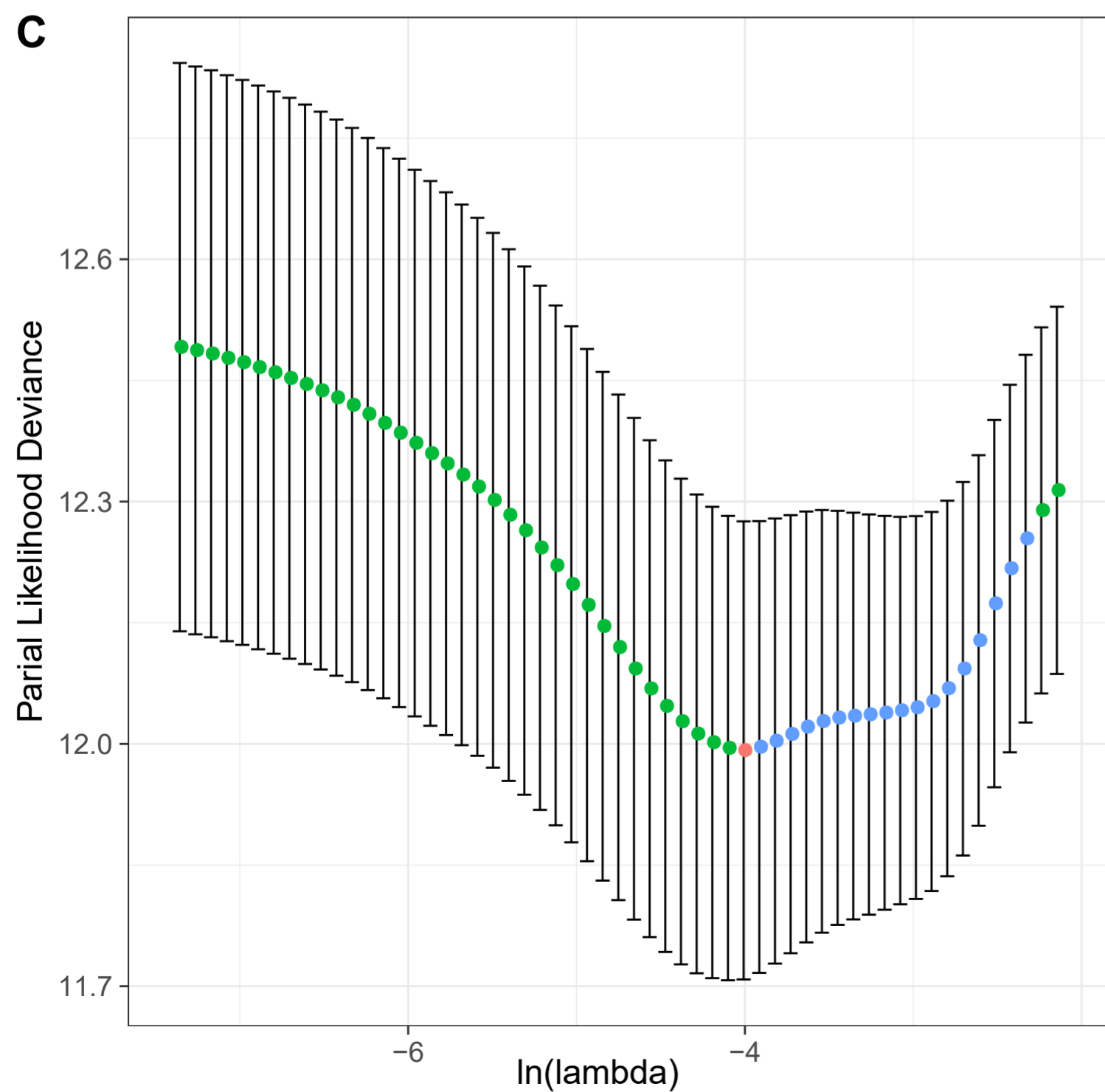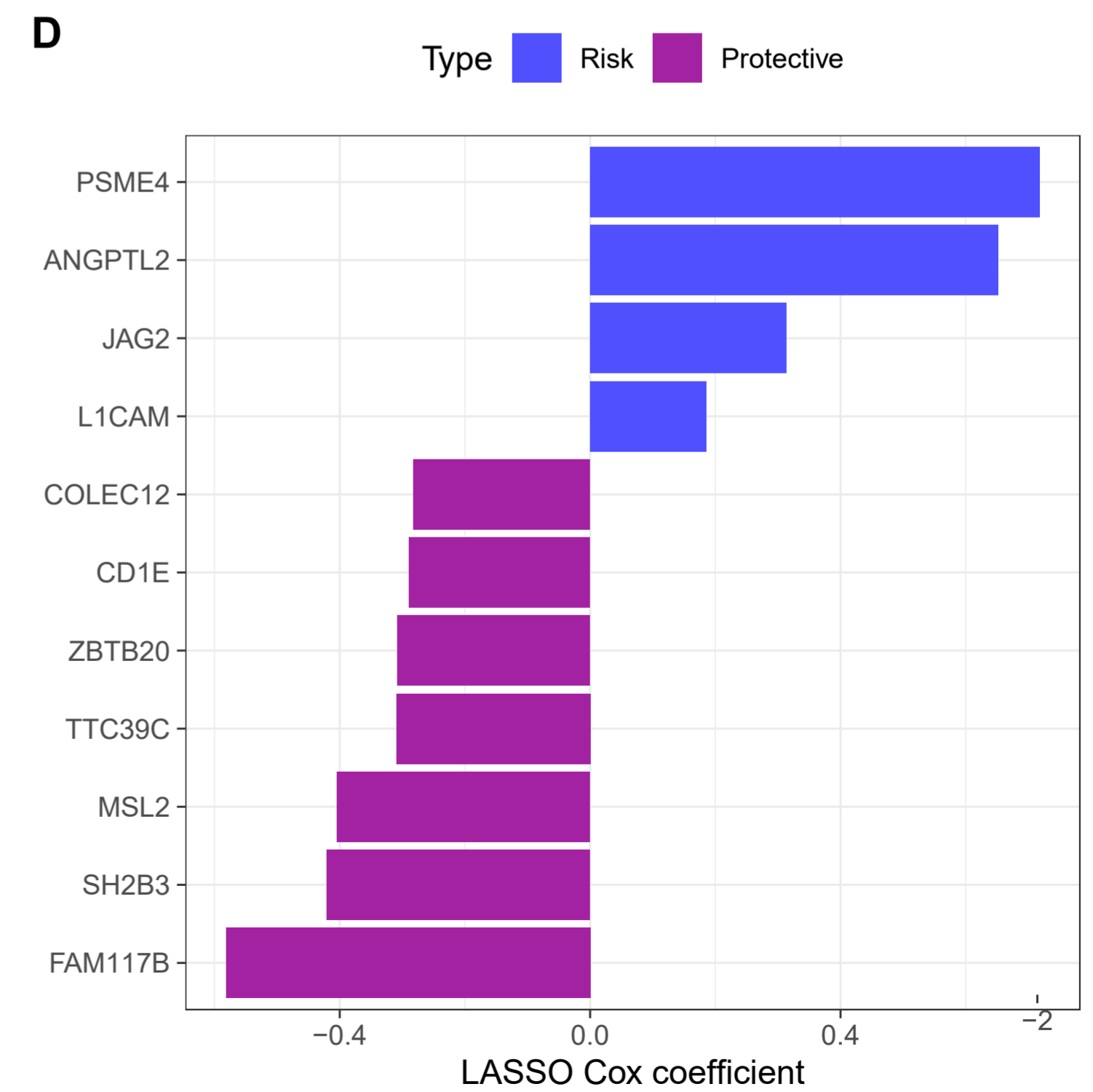

Supplement: Supplemental Information 2 — (A) 40 mRNAs associated with prognosis were screened in Train dataset through univariate Cox regression analysis. (B) 21 mRNAs were found when lambda = 0.0182. (C) The confidence interval under each lambda. (D) 11 mRNAs were identified after stepwise multivariate regression analysis and stepAIC, including 4 risk mRNAs and 7 protective mRNAs. [file peerj-11-15572-s002.pdf]
